# Three decades of a lesson learned from Thailand: compulsory service for dentist workforce distribution

**DOI:** 10.1186/s12960-021-00702-z

**Published:** 2022-01-06

**Authors:** Tanit Arunratanothai, Ravisorn Booncharoen, Sirapop Suwankomolkul, Nareudee Limpuangthip

**Affiliations:** 1 Faculty of Dentistry, Chulalongkorn University, Bangkok, Thailand; 2 Dental Department, Suwannaphum hospital, Roi-et, Thailand; 3 Department of Prosthodontics, Faculty of Dentistry, Chulalongkorn University, 34 Henri-Dunant Rd, Pathumwan, Bangkok, 10330 Thailand

**Keywords:** Admission track, Compensation, Compulsory service, Dentist allocation, Health policy, Health system, Health workforce, Policy implementation

## Abstract

**Background:**

Thailand has encountered an imbalanced dentist distribution and an internal brain drain of dentists from public to private health care facilities. To tackle these challenges, the compulsory service (CS) program, which has been initially implemented for physicians, was extended for dentists.

**Method:**

This policy and workforce document review describes the background, development, and policy implementation of the CS program in Thailand during the past three decades. Outcomes after policy implementation and future directions are also discussed. The information was gathered from the relevant policy and workforce documents available from 1961 to 2021.

**Results:**

In Thailand, junior dentists, specifically newly graduates, have to enroll in the CS program by working as oral health practitioners in public hospitals for at least 3 years. Dentists must pay a maximum fine of 400 000 baht (~ 12 571 USD) if they wish to skip the program. This fine is lowered according to the number of attending years in the program. CS program conditions are related to each university’s admission track. The CS enrolled dentists receive several financial and non-financial benefits, including educational, employment-related, and living provisions. Altogether, successive Thai governments have launched directive policies to increase dentist distribution in rural areas and their retention in public hospitals. These policies have been implemented in 3 stages: (1) increase production of new dentists, (2) allocation of newly dental graduates to public hospitals, and (3) provide benefits for working in public hospitals.

**Conclusion:**

During the past three decades, several public policies have been implemented to improve dentist retention and distribution to public hospitals across Thailand, particularly in rural areas. The present CS program may not completely resolve the oral health inequalities because the dentist retention rate in public hospitals depends on multi-dimensional considerations. Further modifications on the CS program and future well-planned policies are needed.

**Supplementary Information:**

The online version contains supplementary material available at 10.1186/s12960-021-00702-z.

## Background

According to the World Health Organization (WHO), the health workforce is one of the key components, or ‘building blocks’, of the health system. Health workforce allocation should include comprehensive information on the health care personnel, such as the numbers, characteristics, and the location of their hometowns. In addition, this information should include whether those health personnel are currently working and/or studying, and their reasons for resignation from the health sectors [1]. However, a limited health workforce has been one of the major challenges globally and a robust workforce is key to strengthen and ensure proper health care effectiveness inside the health system. These challenges are due to disparities in the distribution, resignation, migration, and death of health care personnel within the health system [1].

To establish the equitable health service accessibility for rural populations, the governments and policy-makers worldwide have to confront the shortage of competent and motivated health workers. According to the WHO estimate, health workforce inadequacy is predominant in low- and middle-income countries, as well as in rural and remote areas. Despite an adequate amount of health workforces, the maldistribution still remains across the country. The issue of health workforce densities and their distribution are emphasized to achieve better health outcomes of people [2].

In order to increase the health workforce distribution and enhance the public accessibility to health services, several countries have employed a compulsory service (CS) program [1]. Thailand is one of them, as it encountered regional and rural imbalances in health workforce numbers and higher resignation rates of health care personnel from public hospitals. Therefore, the Thai government adopted the CS program to resolve these health workforce issues. Initially, the CS program was applied to Thai physicians to tackle the external and internal brain drain phenomenon. Subsequently, the program was extended and adopted for dentists due to increasing oral health inequalities among the Thai population [3–6]. Several directive policies related to the CS program had been implemented and revised according to future policy directions and the social context. However, since the CS program started, its real outcomes and impact on the ground remains fragmented after implementation of several policies. Thus, a comprehensive review of policy and workforce documents is needed for all stakeholders to re-evaluate the CS program outcomes, reconsider the dentist workforce allocation policies, and produce a policy workplan with clear future directions.

The objective of this policy and workforce documentary review was to describe the background, development, and policy implementation of the CS program in Thailand from the start of the program in 1967 until the present date (August 10, 2021). Outcomes following past and current implementation of CS policies and future policy directions were also analyzed and discussed.

### Compulsory service program for Thai dentists

The CS program in Thailand had been implemented for about three decades, initially targeting physicians (1967–1987) and later both physicians and dentists (1988 to the present). The background of the policy development and its action from the past to the present are shown in Table 1. In 1961, the first National Economic and Social Development Plan of Thailand was launched to define the country's strategic plan, which includes a plan for building up the health system. Concurrently, the National Health Plan of Thailand was established, aiming to increase the number of infirmaries across the country [7, 8]. During that time, however, Thailand faced the external brain drain phenomenon because several hundred Thai physicians moved from Thailand to foreign countries for working purposes [3]. The government tackled this challenge by laying down several directive policies to minimize the emigration of physicians and increase their distributions to rural areas where healthcare personnel were lacking.

**Table 1:** Timeline of policy development, affiliates, and its actions

Year	Policy	Rationale	Affiliates	Actions	National Economic and Social Development Plan of Thailand
*1966*	*Propose 2-year compulsory service restriction for physicians* [3]	*Tackle external brain drain*	*Medical school*	*Cabinet disapproved*	1st Plan (1964–1966) Focus on disease prevention and infirmary settlement across countries
*1967*	*1. Establish 3-year compulsory service restriction for physicians* [4]	*Tackle external brain drain*	*MoPH*	*The government partially provided financial subsidies for 6-year undergraduate medical students. Indicate a fine of 200,000 baht if breaking the compulsory service restriction*	2nd Plan (1967–1971) Focus on workforce production and distribution from educational systems to healthcare service, especially in rural areas with difficulty in health service accessibility
	*2. Assign mandated physicians as civil servants* [5]	*Motivate physician retention in public hospitals*	*OCSC*		
*1968*	*1. Propose 3- to 5-year compulsory service program for physicians* [6]	*1. Tackle external brain drain*	*MoPH*	*1. Indicate a 3-year compulsory service program for physicians*	
	*2. Increase physician production in provincial area* [9]	*2. Increase physician production and distribution to rural areas*		*2. Distribute mandate physicians to rural hospitals*	
	*3. Develop central curriculum and examination for all medical institutions* [10]	*3. Control standard of the medical profession*		*3. Establish central examination to obtain a medical license*	
*1973*	*Modify the official compulsory service restriction* [11]	*Tackle external brain drain*	*MoPH*	*Increase fine to 400,000 baht if breaking the compulsory service restriction*	3rd Plan (1972–1976) Increase production and distribution of public transportation to reduce inequalities between urban and rural areas
*1978, 1981*	*Establish the Project of Increased Rural Physician Production* [12, 13]	*Increase physician production and distribution to a rural area*	*NESDC MoPH, MoF*	*Starting point of the project of rural physician production*	4th Plan (1977–1981) Increase workforce production, starting from the educational system, to meet the population's needs
**1982**	**Establish 3-year compulsory service program for dentists** [17]	**Reduce the increasing oral diseases in a rural area**	**MoPH, OCSC**	**All junior dentists of the year 1989 have to enter compulsory service in public hospitals**	5th Plan (1982–1986) Focus on health service systems, from to district levels to subdistricts and villages
		**Increase dentist production and distribution to a rural area**			
		**Tackle external brain drain of the dentist**			
***1987***	***Approve financial welfare for physicians, dentists, and pharmacists*** [27]	***Motivate health workforce retention in public hospitals***	***MoPH, MoF***	***Additional allowance for physicians according to levels of remote area***	6th Plan (1987–1991) Focus on economic development
**1988**	**Allocation of dentists to rural areas** [44]	**Increase dentist distribution to rural areas**	**MoPH, OCSC**	**Allocation of junior dentists to primary care hospitals, public universities, and other state enterprises**	
***1992***	***1. Increase financial compensation for health workforces*** [45]	***1. Motivate health workforce retention***	***MoPH, MoF, BB MoI***	***1. Additional allowance for physicians and dentists (Professional allowance, Shift allowance) and non-private practice allowance only for physicians***	7th Plan (1992–1996) Focus on economic development
	***2. Establish Health Systems Research Institute***	***2. Knowledge management for health system development***	***MoPH***		
***1993***	***Increase financial compensation for health workforces*** [46]	***Motivate health workforce retention***	***MoPH, MoF***	***Additional allowance for dentists and pharmacologists (non-private practice allowance)***	
		***Solving financial injustice in 1992***			
*1994*	*Establish the Collaborative Project to Increase Production of Rural Doctor (CPIRD)* [19]	*Increase physician production and distribution to rural areas*	*MoPH*	*Indicate the criteria for the CPIRD applicants*	
***1997***	***Indicate compensation criteria for the health workforce*** [47]	***Motivate health workforce retention***	***MoPH, MoF***	***Increase allowance for dentists in remote areas***	8th Plan (1997–2001) Focus on economic development
*1999*	*Fulfilled university staff positions*	*Adjust a positioning of university staffs due to an internal education restructure*	*MoPH, MoUA*	*Reposition of civil servants in universities to state enterprise employees*	
***2001***	***Modified the compensation criteria according to the levels of remote area*** [28]	***Motivate health workforce retention in remote areas***	***MoPH, MoF***	***MoPH regulation on the compensation payment 2001 (No.1)***	
***2004***	***1. Modified the compensation criteria according to the level of remote areas***	***Motivate health workforce retention in remote areas***	***MoPH, MoF***	***1. MoPH regulation on the compensation 2001 (revised editions)***	9th Plan (2002–2006) Focus on economic development
	***2. Provided incentive for risking area/South boundary provinces*** [48]	***Motivate health workforce retention***	***MoF, OCSC***	***2. Benefits for the provincial risk areas under the martial law***	
*2005*	*1. The One District One Doctor (ODOD) project* [49]	*1. Increase dentist distribution to rural areas*	*1. MoPH, OCSC*	*1. The ODOD project (12-year CS and 2 million THB fine*	
	***2. The Project of Increased Rural Dentist Production*** [20]	***2. Increase dentist production and distribution to rural areas***	***2. MoPH, MoE***	***2. 10-year project from 2005 to 2014***	
***2005***	***Modified the compensation criteria***	***Motivate health workforce retention*** ***Adjust compensation payment rate and criteria according to each rural area***	***MoPH, MoF***	***MoPH regulations on the compensation payment 2005 (No.2); increase non-private practice allowance***	
***2009***	***Modified the compensation criteria***	***Motivate health workforce retention*** ***Adjust compensation payment rate and criteria according to each rural area***	***MoPH, MoF***	***MoPH regulations on the compensation payment 2009 (No.5): indicate compensation rate of shift allowance, off-site allowance, pay for performance***	10th Plan (2007–2011) Focus on economic development
*2012*	*CPIRD 2013–2017* [50]	*Increase physician distribution to rural areas*	*MoE, SoC*	*CPIRD 2013–2017*	11th Plan (2012–2016) Focus on economic development
***2013***	***Modified the compensation criteria***	***Motivate health workforce retention***	***MoPH, MoF***	***MoPH regulations on the compensation payment 2013 (No.8): indicate non-private practice allowance***	
***2013***	***Modified the compensation criteria***	***Motivate health workforce retention***	***MoPH, MoF***	***MoPH regulations on the compensation payment 2013 (No. 9): specify the criteria, methods, and conditions of compensations***	
***2016***	***Modified the compensation criteria***	***Motivate health workforce retention*** ***Adjust compensation payment rate and criteria according to each rural area***	***MoPH***	***MoPH regulations on the compensation payment (No. 11): specify the criteria, methods, and conditions of remote area allowance***	

CS, compulsory service; OCSC, Office of Civil Service Commission; MoE, Ministry of Education; MoF, Ministry of Finance; MoPH, Ministry of Public Health; MoUA, Ministry of University Affairs; NESDC, Office of the National Economic and Social Development Council; SoC, the Secretariat of the Cabinet

Health workforce includes physicians, dentists, and pharmacists

Noted that italic indicates the policies directly related to medicine; bold indicates the policies directly related to dentistry; and bolditalics indicates the policies related to medicine, dentistry and pharmaceutical sciences.

In 1967, the official directive policy was launched including enforcement measures dictating that all medical students should receive financial subsidies from the Thai government during their 6-year undergraduate education. Therefore, the newly medical graduates had to enroll in the CS program as mandated physicians in public hospitals for 3 years [4]. Later on, a fine for not properly enrolling into the program, including the refusal or early resignation from the CS program, was strongly implemented [5].

In 1968, the number of medical schools and medical students per year was raised to increase the physician production rate. Public hospitals also increased to support mandated physicians [9]. To control medical profession standards, core curriculum and central examination were applied to all institutions [10]. In 1973, a maximum fine increased from 200 000 to 400 000 baht (~ 6285 to ~ 12 571 USD) [11]. This fine was lowered according to the number of attended years in the CS program.

In 1978, the government established the “Project of Increased Rural Physician Production” (PIRPP) to increase the physician distribution in rural areas [12, 13]. Data reported that the proportion of physicians completing the 3-year CS program were more likely from this project than from other application tracks. We believe this had occurred because the PIRPP project included additional restrictions in the CS program such as an option to select predetermined provinces for compulsory work that were somehow linked with the hometown of the applicants (e.g., nearby provinces adjacent to their home residence) [14–16].

In 1982, a similar policy was applied to newly dental graduates. This enforcement for Thai dental students was established upon the condition that they would receive financial subsidies partially provided by the government during their 6-year undergraduate education. Therefore, the newly qualified dental graduates would have to enroll in the CS program in public hospitals for at least 3 years. Otherwise, newly graduates would be fined 400 000 baht as a form of reimbursement, and such fine would be adjusted according to the number of attended years in the CS program alike the policies for the medical graduates [17, 18].

During a period of sustained economic growth in Thailand (~ 1987–1996), several private health care facilities had emerged. The government encountered at this time “internal brain drains” from public to private health care facilities. During 1994–1995, the “Collaborative Project to Increase Production of Rural Doctors (CPIRD)” was established to increase the retention rate of mandated physicians in rural areas [19]. In 2005, the government extended the CPIRD model to dentists to increase their production numbers and distribution to rural areas, and called this one the “Project of Increase Rural Dentist Production”, which was approved and implemented for a period of 10 years (2005–2014) [20, 21].

Since dentist workforce production is linked to the educational output from Thai universities, the academic admission process should be analyzed for allocating and distributing the newly dental graduates.

### Dental workforce production systems

Dental workforce production in Thailand was officially implemented in 1940 by the first Faculty of Dentistry in the Kingdom which was created at Chulalongkorn University. To date, there are 16 dental schools (12 public and 4 private schools) distributed across 4 regions; 4 Northern, 2 North-eastern, 1 Southern, and 9 Central, covering Bangkok and adjacent provinces.

Some differences between public and private dental schools are noted. Because public dental schools receive financial support partially from the government, their tuition fee is lower and more fundings are offered to the students in public dental schools compared with those in private dental schools. Meanwhile, students in private dental schools tend to afford educational and living expenses. In addition, the strategies of public dental schools rather depend on and follow the government policies such as the number of new students and admission tracks.

The undergraduate dental education in Thailand offers a 6-year curriculum with the Doctor of Dental Surgery (DDS) degree. Preclinical courses must be completed within the first 3 years, and clinical courses start on the 4th and end on the 6th year. To obtain a dental practicing license, all dental undergraduates must pass a national standard examination [22].

#### University admission tracks and compulsory service conditions

**Fig. 1:**
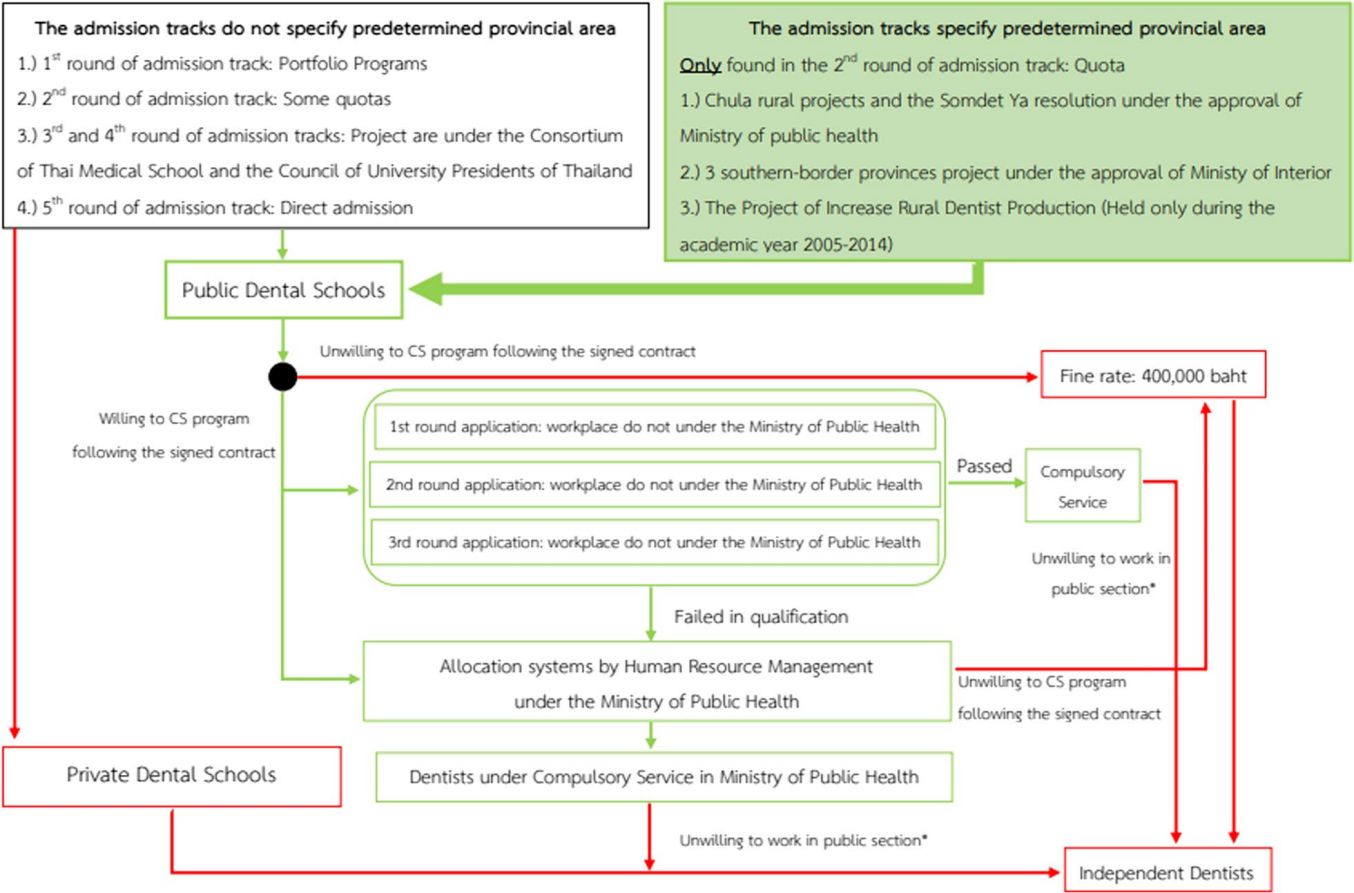
Flowchart of universities admission tracks and CS program conditions in Thailand. Note: *Paying the fine, of which the rate is lowered according to the number of attended years in the CS programs. Green and red arrows indicate the pathways into and out of CS program, respectively. Green and red outline boxes indicate the process in the public and private sectors, respectively. Green shading box indicates the admission process that has a positive impact on retention in CS program

The admission system of the undergraduate dental curriculum in Thailand had been constantly modified following educational policies and different socio-economic and political contexts. At present, there are 5 admission tracks with some differences or specific requirements between universities and faculties [23]. Except for private dental schools, government directive policies state that graduate newly dental graduates from all tracks have to enroll in the CS program as mandated dentists in public hospitals for a period of 3 years (Fig. 1).

The 1st round is the Portfolio submission, and it targets the especially talented applicants with specific skills in English language and other required scientific backgrounds for enrolling in Dentistry (i.e., Biology and Chemistry). As an example, the Intensive English Admission Project and the International Science Olympiads Admission Project [24]. The 2nd round establishes the Quota, and it includes the applicants with specific requirements taking into consideration the location of their hometown. The quotas include additional restrictions for the CS program. For example, the Chula-rural project under the Somdet Ya resolution (elaborated to appraise the Royal programs and efforts) recommends a 12-year working duration in public hospitals, and meanwhile, the Chula-rural project requires the existence of predetermined provinces for working after graduation [25]. The goal here is to increase the retention of dentists in rural areas.

The 3rd round (Admission I) and the 4th round (Admission II) are under the responsibility of the Consortium of Thai Medical School (COTMES) and the Council of University Presidents of Thailand, respectively. Nationwide applicants can choose 10 faculties of interest and can enter into the highest-ranking faculty if they achieve a minimum score to enroll. The 5th (final) round is a Direct Admission one, which includes nationwide applicants and aims to fulfill the vacant seats after the 4th round [23].

Among all the five tracks, the quota track is the only university admission track created to improve dentist workforce distribution and retention in rural areas.

#### Work routes for newly dental graduates

Three employability routes are available for newly dental graduates according to the following categories:

1. Civil servant: this route is available for all junior dentists from public dental schools who enter the CS program in public hospitals under the Ministry of Public Health (MoPH). Two allocation systems are available according to their admission tracks. The junior dentists from the quota admission track undergo the CS program in the predetermined provincial area, while those from the other admission tracks are allocated to the working area by drawing and casting lots. The latter is a majority of the junior dentists.
  The drawing and casting lots are held annually around May by the MoPH. Before 2020, the allocation system was commonly held physically in convention centers. During the COVID-19 pandemic in 2020–2021, the allocation process was changed to a virtual platform. This was the second time after 2006, the first and only time of the virtual allocation process.
2. State enterprise employee: this employability option is available for all junior dentists from public dental schools who enter CS program in public hospitals which are not under the supervision of MoPH, such as academic and military institutions. The enrollment, commonly taken during the last academic year of the dental curriculum, includes direct application and a face-to-face interview conducted by staff from those institutions.
3. Independent dentists: this option is open for all newly dental graduates from private schools who do not have to pay the CS program-related fine (because they were not offered the CS program). Meanwhile, the newly dental graduates from public schools who are unwilling to enter the CS program must pay a fine (400 000 Baht or ~ 12 571 USD) to their dental schools as they received a financial subsidy from the Thai government during their entire undergraduate education.

Moreover, it is important to note that the Thai government provides extra welfare benefits in addition to the monthly salary to further increase the retention of civil servants. Meanwhile, welfare for state enterprise employees has specific differences depending on the granting institutions.

### Benefits for oral health practitioners in public hospitals

Globally, there are 3 types of CS programs: (1) the state employment program, (2) CS program without incentives, and (3) CS program with incentives [26]. CS program with bundled incentives is adopted in Thailand, including educational, employment-related, and living provisions. Both state employees and civil servants working in public hospitals can be under the supervision of different institutions. Thus, different employment benefit packages are offered to dentists according to each institution (Table 2).

**Table 2:** Comparisons of benefit packages between civil servant, state enterprise employee, and private employee

	Civil servant	State enterprise employee	Private employee
Wage	✓	✓	✓
Professional Allowance	✓	✓	✘
Compensation for rural area	✓	✘	✘
Educationally	✓	✘a	✘
Living provision	✓	✘	✘
Health insurance	CSMBS	SSS	UCS

a Depends on the affiliate. CSMBS, Civil servant medical benefit scheme; SSS, Social security scheme; UCS, Universal coverage scheme

Benefits for civil servants include salary and welfare, comprising financial and non-financial compensations. In addition to the monthly salary, 4 types of financial welfare exist: (1) a professional allowance that increases depending on the highest educational degree of the civil servant; (2) a non-private practice allowance, a fixed amount for dentists who do not carry out private practice after regular working hours; (3) an allowance for dentists in rural areas that is different across the provincial areas (certain provinces will have extra benefits according to government policies for that specific fiscal year) and (4) a shift allowance that relies on either the working duration or hours of workload [27, 28].

Non-financial compensation for civil servants includes higher education, employment, and living provision. Oral health practitioners in public hospitals who leave for study still receive a monthly salary during study duration, but after graduation, they are required to go back to work at those affiliated hospitals for a specific amount of time. Other employment incentives include free dormitory, financial support for their children’s tuition, and a civil servant medical benefits scheme that covers not only the civil servants, but their family members also [29, 30].

## Discussion

Successive Thai governments have launched several directive policies to increase the distribution of dentists in rural areas and augment their retention in public hospitals. We categorized the implementation of such policies into 3 stages: (1) increase production of new dentists nationwide, (2) allocation of newly dental graduates to public hospitals, and (3) enhancement of benefits for working in public hospitals. These strategies and their respective outcomes are discussed below.

### Increase production of new dentists nationwide

**Fig. 2:**
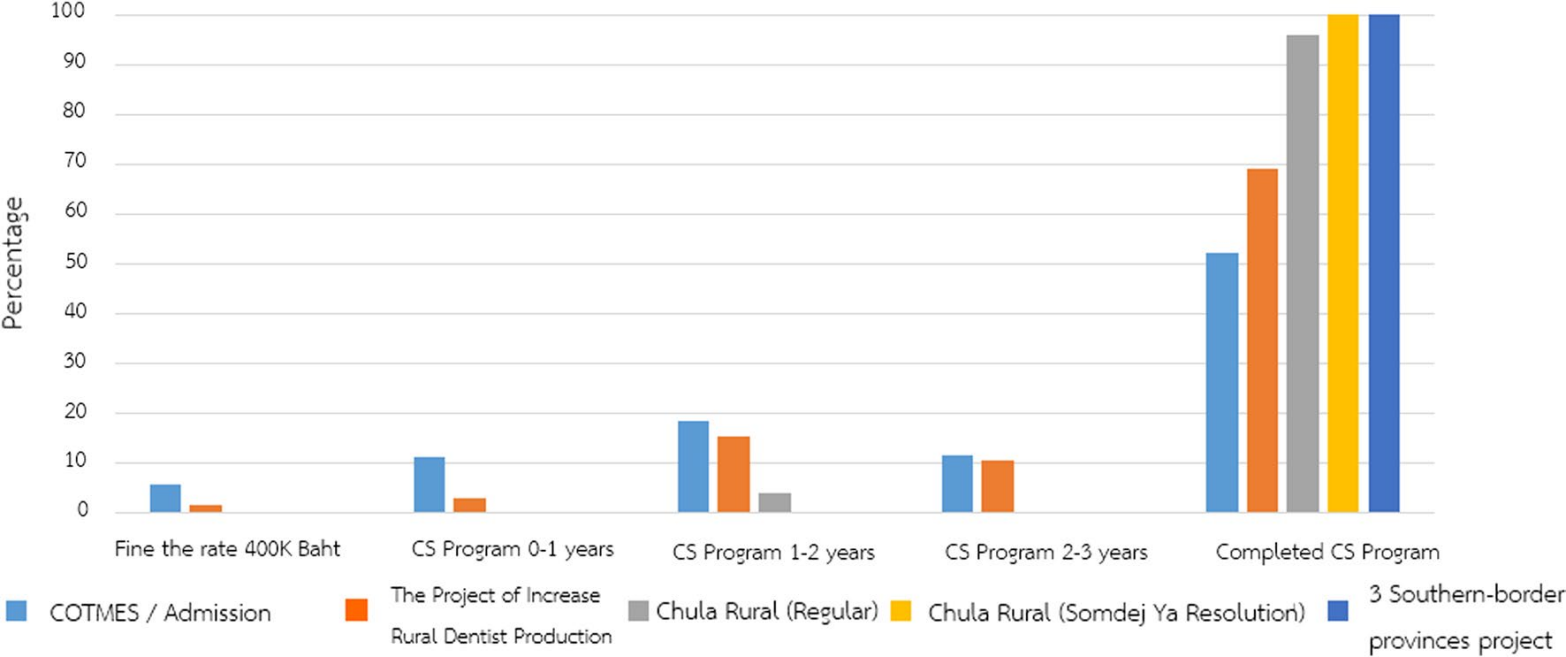
Percent retention in CS program of dentists who graduated in academic year 2015–2017 from DDS program, Faculty of Dentistry, Chulalongkorn University (different colors indicate different admission tracks)

**Fig. 3:**
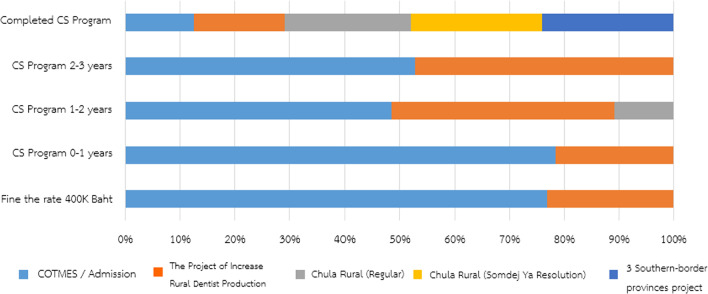
Percent resignation from compulsory service program between admission tracks of dentists who graduate from DDS program, Faculty of Dentistry, Chulalongkorn University in academic year of 2015–2017 (different colors indicate different admission tracks)

Each university admission track establishes the conditions of Dentistry applicants and future graduates to enter the CS program. The future dentists from the quota track must undergo CS program in the predetermined provinces, commonly located around their hometown places. Recent compiled data from Academic Affairs at Faculty of Dentistry, Chulalongkorn University showed that the dentists arising from the “Quota admission track” completed the full 3-year CS working program in higher proportion rates and had lower resignation rates during the first working year, when compared with other admission tracks (Figs. 2 and  3). This data is consistent with data from medical graduates and it indicates that the “Quota admission track” tends to reduce the disparities in the number of dentists across the country [31, 32].

### Allocation of junior dentists to public hospitals

The public institutions recruit junior dentists to become state employees via face-to-face interviews during the last academic year of undergraduate education. Then, the MoPH allocates junior dentists to become civil servants at the end of the academic year by drawing and casting lots. Thus, these graduates may not get to work in their expected areas in the CS program due to a potentially limited quota for each province.

According to CS program data from the Human Resource Management Department under MoPH, the dentist allocation process by drawing lots through an online platform showed that 25.6% and 26.2% of newly dental graduates failed in getting to work on their expected areas in 2020 and 2021, respectively. In addition, 0.60% and 1.13% of them, in 2020 and 2021, respectively, refused to enter the CS program and were willing to pay the fine [33, 34]. A further longitudinal study is needed to identify whether the newly dental graduates in the CS program who get to work in their expected locations of interest have longer retention rates than those who were not so lucky with the draw.

### Benefits for working in public hospitals

An intense policy enforcement for newly medical graduates who decided not to enter the CS program was proposed in 1968 and 1973, including a higher fine and the completion of the full 3-year CS program. These medical graduates were able to receive a graduation certificate and passport after the 3-year CS program, though an early resignation would result in the withdrawal of the medical license [35]. At present, however, the only official directive and enforced policy mandates a maximum fine of 400 000 baht (~ 12 571 USD) if not attending the CS program. To increase dentist retention in public hospitals, several financial and non-financial compensations are also provided for oral health practitioners.

These directive measures and working compensation schemes may help increase the number of dentists in rural areas. However, an unequal dentist distribution between public and private health care facilities, between Bangkok and other regions, and between urban and rural areas persist for the last three decades [36–38]. These shortcomings should carefully be analyzed to tackle the root of these oral health inequalities. Regardless of the selected academic admission track, any newly dental graduate can choose to pay the fine to drop out of the CS program. Therefore, the “quota track”, which is the only admission track created to reduce imbalances on the dentist workforce across the country, can be circumvented and this generates important shortcomings in tackling oral health inequalities. The CS program’s drop out or resignation fine should have been revised once every 5 years following real socio-economic changes, however, such fines remained the same during the last 32 years. If one assumes an average Thailand inflation rate for the last 32 years (BE 2532–2563) at 2.61% [39], then a 2.10 inflation coefficient value should be computed, and the maximum fine should have been 840 000 baht (400 000-baht × 2.10) or ~ 25 142 USD at the present time (Additional file 1: Table S1). However, additional adjustments to the fine may be necessary after considering other factors such as academic tuition fee, financial support from the government/academia, and opinions of the different universities and hospitals.

Despite the existence of provisions by several public welfare schemes, the monthly income of Thai oral health practitioners in public hospitals is approximately 40.9–59.7% lower than those in private practice [40]. Dentists in public hospitals commonly have an additional workload that goes beyond their clinical responsibilities, such as hospital management and excessive administration bureaucracy along the decision-making chain. Meanwhile, private dentists can stay focus mainly on their oral health care responsibility. Moreover, the government disbursement system for dentists in public hospitals is relatively delayed especially the monthly allowance as it mainly depends on the financial liquidity of each local hospital; conversely, disbursement of allowance for dentists in private hospitals are generally punctual. Since income is one of the major employment concerns among junior dentists, disparities between income and ultimate workload affects dentists’ early resignation from public hospitals [41].

In addition to financial welfare, access to higher education is also one of the factors negatively affecting dentist retention rates in public hospitals. The Thai government supports only a monthly income for CS-mandated dentists during their enrollment in continuing education programs, ignoring tuition expenses borne by junior dentists. In addition, types of dental specialties for the purpose of continuing education are limited by directive management policies of local hospitals. Consequently, junior dentists often resign from public hospitals or change their hospital affiliation whenever their continuing education aspirations do not meet their employer’s policies. Unlike in medical programs, several postgraduate dental education programs do not impose applicants to have a public institution affiliation and/or working experience in public hospitals as prerequisites to enter in such programs. Thus, the current educational benefits scheme may not be sufficient to attract the junior dentists and retain them in public hospitals.

### Future workplan and directions to improve the CS program

The dentist workforce production strategy should consider not only a target number of newly graduated dentists per year, but also specific characteristics that meet the requirements and demands of the different stakeholders. This strategy will ultimately make such dentist distribution more equal across the country. Establishing dental labor market needs by committees would be essential in order to annually identify the number and criteria of undergraduate dental applicants, as well as to improve the predoctoral and postdoctoral curriculum to fulfill market and public health demands. This labor market committee would be responsible for setting up a dentist production plan for a 6-year time frame (as per current predoctoral curriculum duration), which could be adjusted by dental schools. Since newly dental and medical graduates prefer selecting workplaces that are located close to their hometowns [14, 15, 42], determining such workplace locations will have an impact on their retention by public hospitals. Universities may also have an important role in this strategy and should consider increasing the number of students in the “Quota admission track” to augment their retention in public hospitals and promote an even distribution between urban and rural areas. This can be achieved through the CS program because they have to undertake such program in public hospitals located in their hometowns, or in rural areas nearby or in adjacent provinces.

Determining underlying causes for dentists’ retention or resignation from public hospitals is necessary to set up policies for the various stakeholders. According to Herzberg's Motivation Theory [43], working motivation is divided into hygiene and motivation factors. Motivation factors, including work success, acceptability, and work progress directly affect working. Meanwhile, hygiene factors including welfare, medical benefits, and interpersonal relations, do not lead to positive motivation in the long term, however, they can prevent dissatisfaction. Presently, the established public policy increases dentist retention by promoting hygiene factors and medical benefits. Further directions should include a policy that promotes motivation factors to improve long-term dentist retention in public hospitals.

## Conclusions

During the past three decades, several public policies have been established and implemented to improve dentist retention and distribution to public hospitals across Thailand, particularly in rural areas. The present CS program may not completely resolve the oral health inequalities because the dentist retention rate in public hospitals depends on multi-dimensional considerations. Further modifications on the CS program and future well-planned policies are needed in Thailand for proper oral health workforce development, consistent rural distribution, and long-term retention in public health system.

## Supplementary Information

**Additional file 1: Table S1.** Inflation rate of Thailand and inflation coefficient value.

## Data Availability

All data sets generated or analyzed during this study are included in the published article and the Additional file 1.

## Abbreviations

COTMES: Consortium of Thai Medical School
CPIRD: Collaborative Project to Increase Production of Rural Doctors
CS: Compulsory service
CSMBS: Civil servant medical benefit scheme
DDS: Doctor of Dental Surgery
MoE: Ministry of Education
MoF: Ministry of Finance
MoPH: Ministry of Public Health
MoUA: Ministry of University Affairs
NESDC: Office of the National Economic and Social Development Council
OCSC: Office of Civil Service Commission
PIRPP: Project of Increased Rural Physician Production
SoC: Secretariat of the Cabinet
SSS: Social security scheme
UCS: Universal coverage scheme
WHO: World Health Organization
