# Supplementary material for: Three decades of a lesson learned from Thailand: compulsory service for dentist workforce distribution
Source: Hum Resour Health. 2022 Jan 6;20:5. doi: 10.1186/s12960-021-00702-z (PMC8733760; doi:10.1186/s12960-021-00702-z)
Supplement: Supplementary file 1 — Additional file 1: Table S1. Inflation rate of Thailand and inflation coefficient value. [file 12960_2021_702_MOESM1_ESM.docx]

**Additional file 1**

**Table S1.** Inflation rate of Thailand and inflation coefficient value.

$$Fine Rate at 2021 = Fine Rate at 1989 x inflation coefficient value$$

| Inflation coefficient value = (1 + inflation rate)^years^ | | | | | | | | |
| --- | --- | --- | --- | --- | --- | --- | --- | --- |
| inflation  rate | Years | | | | | | | |
| (%) | 0 | 5 | 10 | 15 | 20 | 25 | 30 | 35 |
| 0.0 | 1.00 | 1.00 | 1.00 | 1.00 | 1.00 | 1.00 | 1.00 | 1.00 |
| 0.5 | 1.00 | 1.03 | 1.05 | 1.08 | 1.10 | 1.13 | 1.16 | 1.19 |
| 1.0 | 1.00 | 1.05 | 1.10 | 1.16 | 1.22 | 1.28 | 1.35 | 1.42 |
| 1.5 | 1.00 | 1.08 | 1.16 | 1.25 | 1.35 | 1.45 | 1.56 | 1.68 |
| 2.0 | 1.00 | 1.10 | 1.22 | 1.35 | 1.49 | 1.64 | 1.81 | 2.00 |
| 2.5 | 1.00 | 1.13 | 1.28 | 1.45 | 1.64 | 1.85 | 2.10 | 2.37 |
| 3.0 | 1.00 | 1.16 | 1.34 | 1.56 | 1.81 | 2.09 | 2.43 | 2.81 |
